# Microbial Metabolic Capacity for Intestinal Folate Production and Modulation of Host Folate Receptors

**DOI:** 10.3389/fmicb.2019.02305

**Published:** 2019-10-09

**Authors:** Melinda A. Engevik, Christina N. Morra, Daniel Röth, Kristen Engevik, Jennifer K. Spinler, Sridevi Devaraj, Sue E. Crawford, Mary K. Estes, Markus Kalkum, James Versalovic

**Affiliations:** ^1^Department of Pathology and Immunology, Baylor College of Medicine, Houston, TX, United States; ^2^Department of Pathology, Texas Children’s Hospital, Houston, TX, United States; ^3^Integrative Molecular and Biomedical Sciences, Baylor College of Medicine, Houston, TX, United States; ^4^Department of Molecular Imaging and Therapy, Beckman Research Institute of the City of Hope, Duarte, CA, United States; ^5^Department of Molecular Virology and Microbiology, Baylor College of Medicine, Houston, TX, United States; ^6^Department of Medicine – Gastroenterology, Hepatology and Infectious Diseases, Baylor College of Medicine, Houston, TX, United States; ^7^Mass Spectrometry and Proteomics Core, Beckman Research Institute of the City of Hope, Duarte, CA, United States

**Keywords:** B vitamin, enteroids, folate transporters, folylpolyglutamate, *Lactobacillus reuteri*, Lactobacilli, microbiome

## Abstract

Microbial metabolites, including B complex vitamins contribute to diverse aspects of human health. Folate, or vitamin B_9_, refers to a broad category of biomolecules that include pterin, para-aminobenzoic acid (pABA), and glutamate subunits. Folates are required for DNA synthesis and epigenetic regulation. In addition to dietary nutrients, the gut microbiota has been recognized as a source of B complex vitamins, including folate. This study evaluated the predicted folate synthesis capabilities in the genomes of human commensal microbes identified in the Human Microbiome Project and folate production by representative strains of six human intestinal bacterial phyla. Bacterial folate synthesis genes were ubiquitous across 512 gastrointestinal reference genomes with 13% of the genomes containing all genes required for complete *de novo* folate synthesis. An additional 39% of the genomes had the genetic capacity to synthesize folates in the presence of pABA, an upstream intermediate that can be obtained through diet or from other intestinal microbes. Bacterial folate synthesis was assessed during exponential and stationary phase growth through the evaluation of expression of select folate synthesis genes, quantification of total folate production, and analysis of folate polyglutamylation. Increased expression of key folate synthesis genes was apparent in exponential phase, and increased folate polyglutamylation occurred during late stationary phase. Of the folate producers, we focused on the commensal *Lactobacillus reuteri* to examine host–microbe interactions in relation to folate and examined folate receptors in the physiologically relevant human enteroid model. RNAseq data revealed segment-specific folate receptor distribution. Treatment of human colonoid monolayers with conditioned media (CM) from wild-type *L. reuteri* did not influence the expression of key folate transporters proton-coupled folate transporter (PCFT) or reduced folate carrier (RFC). However, CM from *L. reuteri* containing a site-specific inactivation of the *folC* gene, which prevents the bacteria from synthesizing a polyglutamate tail on folate, significantly upregulated RFC expression. No effects were observed using *L. reuteri* with a site inactivation of *folC2*, which results in no folate production. This work sheds light on the contributions of microbial folate to overall folate status and mammalian host metabolism.

## Introduction

The human gut microbiota harbors a complex and dynamic microbial community that is dominated by Firmicutes and Bacteroidetes, with a lesser proportion of Actinobacteria, Proteobacteria, Fusobacteria, and Verrucomicrobia (D’Argenio and Salvatore, 2015). Collectively the intestinal microbiota plays a pivotal role in nutrient digestion and production of host-modulating metabolites (O’Connor, 2013; Berding and Donovan, 2016; Maruvada et al., 2017). The fermentation and metabolic conversion of dietary components by intestinal bacteria results in diverse metabolites such as SCFAs, long chain fatty acids, lactic acid, and biogenic amines (Cummings and Macfarlane, 1997; Kimura et al., 2002; Hayakawa et al., 2004; Izquierdo Canas et al., 2009; Tabanelli et al., 2012; Thomas et al., 2012; Frei et al., 2013; Ferstl et al., 2014; Hollister et al., 2014; Kibe et al., 2014; Luk et al., 2018; Shimizu et al., 2018). These metabolites may have an impact on the intestinal milieu and mammalian health status. Vitamins are another such end-product (Birnbaum et al., 1967; Friend et al., 1983; Cummings and Macfarlane, 1997; Hugenholtz and Smid, 2002; Crittenden et al., 2003; Streit and Entcheva, 2003; Burgess et al., 2006; Jayashree et al., 2010; LeBlanc et al., 2011, 2013b). Vitamins cannot be synthesized by mammals and therefore must be obtained through intestinal absorption from exogenous sources, including dietary sources and the gut microbiota (Rossi et al., 2011). This point is clearly demonstrated by the fact that germ-free animals lacking a microbiota require supplementation of vitamin K and certain B vitamins, while their conventionalized complete microbiota counterparts do not require these vitamins (Rossi et al., 2011). Bacterial genera common in the distal intestine, including *Bacteroides*, *Bifidobacterium*, and *Enterococcus*, are known to synthesize vitamins (Morowitz et al., 2011). Previous studies have identified individual species capable of producing vitamins (Birnbaum et al., 1967; Friend et al., 1983; Cummings and Macfarlane, 1997; Hugenholtz and Smid, 2002; Crittenden et al., 2003; Streit and Entcheva, 2003; Burgess et al., 2006; Jayashree et al., 2010; LeBlanc et al., 2011, 2013b). Additionally, several studies have performed in-depth analyses of vitamin production by microbes from the human intestine (Magnusdottir et al., 2015; Rowland et al., 2018; Das et al., 2019) These studies have highlighted the need for an evaluation of the folate synthesis capabilities of a broad cohort of bacterial strains, in conjunction with a detailed evaluation of the folates synthesized, as well as the specific effects of those folates on the human intestine.

Microbes produce B complex vitamins, including B_1_, B_6_, B_12_, and vitamin B_9_. Folate, or vitamin B_9_, is a broad category of biomolecules characterized by containing a complex of pterin, pABA, and glutamate subunits (Blakley and Benkovic, 1984). Folate biosynthesis requires a complex pathway involving 16 enzymatic processes (KEGG, 2017; Kanehisa and Goto, 2000). First, chorismate is produced from the glycolysis product, phosphoenolpyruvate which combines with pABA. GTP is converted to dihydropterin pyrophosphate prior to combining pABA and dihydropterin pyrophosphate intermediates. Finally, glutamylation and reduction yield THF, the simplest form of bioavailable folate (Scott, 1999; Rossi et al., 2011). The majority of naturally occurring folates are THF, 5-methyl THF, and 10-formyl THF, which are polyglutamylated. Bacteria synthesize mono- and poly-glutamylated folate (also known as 5,10-methenyl- THF or FPG), folate forms that are easily processed and absorbed by mammalian cells (Kim T. H. et al., 2004; Thomas et al., 2016). Microbe-generated vitamins, including folate, are primarily absorbed in the colon, while dietary vitamins are largely absorbed in the small intestine (Ichihashi et al., 1992; Said and Mohammed, 2006). Several bacterial strains within the human microbiome synthesize and secrete THFs which can be absorbed by the intestinal epithelium (Camilo et al., 1996; Asrar and O’Connor, 2005; Maynard et al., 2018). Previous studies have evaluated potential probiotic strains such as *Bifidobacterium* and *Lactobacillus* as a source of folate compounds (LeBlanc et al., 2011, 2013b). However, few studies have examined the capacity of the gut microbiome to generate folate. Moreover, a paucity of data have been published defining microbe-mediated folate modifications, such as polyglutamylation.

On the host side, dietary and microbe-produced folate uptake occurs through four proteins: folate receptor alpha (FOLR1), folate receptor beta (FOLR2), PCFT, or RFC (Zhao and Goldman, 2007; Visentin et al., 2014). FOLR1 and FOLR2 are localized to tissue outside the intestine and neither receptor is believed to contribute significantly to intestinal folate absorption (Hague et al., 1995; Barber et al., 1999; Kim, 1999, 2006; Kamen and Smith, 2004; Kim D. H. et al., 2004; Blom et al., 2006; Pitkin, 2007; Vollset et al., 2013; Wibowo et al., 2013; Zhao and Goldman, 2013; Visentin et al., 2014; Shen et al., 2015; Balashova et al., 2018). In contrast PCFT and RFC folate transporters are postulated to play a significant role in intestinal folate transport (Visentin et al., 2014). To the best of our knowledge, no study has examined folate transporter distribution along the length of the human intestine or identified folate transport/assimilation mechanisms in different regions of the gastrointestinal tract. Existing studies examining folate receptor expression and function have relied on cancer-derived cell lines. However, intestinal cancer cells exhibit a hallmark upregulation of FOLR1, which is not normally present in the healthy colon and this upregulation may affect other folate transporter responses. Another major gap in knowledge is the lack of studies describing folates produced by microbes and mammalian folate transporters. A more accurate model of the intestine is needed to identify folate-related functions in response to microbial folate compounds.

Folate is essential for several metabolic processes including one-carbon transfer, methylation metabolism through the synthesis of SAM, and the synthesis of thymidylate, purines, and several amino acids (Baggott and Tamura, 2015; Brosnan et al., 2015). Once absorbed, folate also participates in nucleotide synthesis and replication, repair, and methylation of DNA. Given the important role of folates in cell maintenance, deficiency in folate has been linked to a large spectrum of health disorders including coronary heart disease, osteoporosis, Alzheimer’s disease, and colorectal cancer (LeBlanc et al., 2011, 2013a). Bacterial folate has been put forward as a potential nutrition source for treating or preventing low-folate conditions (Pompei et al., 2007b; Rossi et al., 2011). However, this effect has been hindered by the lack of characterization of folate-secreting intestinal bacterial strains.

In this study, the predicted folate synthesis capabilities of representative members of the human intestinal microbiome were evaluated to determine the patterns and prevalence of bacterial folate metabolism genes. Bacterial folate production was examined in a phylogenetically diverse set of intestinal microbes representing six key phyla of the human intestinal microbiome. Additionally, a more in-depth characterization of folate glutamylation by two key commensal microbes was examined by mass spectrometry. To connect microbe-derived folate metabolism with uptake by the host, we assessed folate-associated processes in human intestinal enteroids by RNAseq and identified the potential for microbial folate compounds and FPGs to alter the expression of folate transporters in human enterocytes.

## Materials and Methods

### Bacterial Strains and Culture Conditions

All bacterial strains and growth conditions used in this study are described in Supplementary Table S1. Bifunctional dihydrofolate synthase/folylpolyglutamyl synthase type 2 (folC2) and bifunctional dihydrofolate synthase/folylpolyglutamyl synthase (folC) genes (GenBank NZ_ACGX02000001−007, HMPREF0536_11260, and HMPREF0536_10555, respectively) were inactivated in *Lactobacillus reuteri* ATCC PTA 6475 (*L. reuteri* 6475) by site-specific integration of plasmid pORI28 into the *L. reuteri* 6475 chromosome as described previously (Walter et al., 2005; Thomas et al., 2016). Dideoxy DNA sequencing was used to confirm site-specific insertional mutagenesis. *L. reuteri* 6475, *L. reuteri* 6475 *folC2*:pORI28 (referred to as folC2), and *L. reuteri* 6475 *folC:*pORI28 (referred to as *folC)* were cultured in MRS medium. Insertion mutants were cultured in the presence of 10 μg/mL erythromycin. *Bifidobacterium longum* subspecies *infantis* ATCC 15697 was also cultured in MRS medium. *Prevotella copri* CB7 DSM 18205 and *Clostridium sporogenes* DSM 795, ATCC 3584 were grown in brain–heart-infusion (BHI) broth supplemented with 2% yeast extract and 0.2% cysteine. *Akkermansia muciniphila* ATCC BAA-835 was cultured in BHI supplemented with 0.4% pig stomach mucin (Sigma Aldrich). *Escherichia coli* Nissle 1917 was cultured in Luria-Bertani (LB) broth. All cultures were grown in an Anaerobe Systems AS-580 anaerobic chamber supplied with a mixture of 10% CO_2_, 5% H_2_, and 85% N_2_. Following initial growth, cultures were subcultured into the appropriate fully defined medium. Bacterial media recipes and reagents for the fully defined media are labeled as LDM4 and CDMM and compiled in Supplementary Tables S2, S3, respectively.

For growth curves, cultures of *L. reuteri* 6475, *L. reuteri* 6475:*folC2*, and *L. reuteri* 6475:*folC* grown at exponential phase in MRS were used to inoculate the fully-defined medium LDM4 (OD_600__nm_ adjusted to 0.1) and incubated anaerobically at 37°C. Growth was examined by optical density (OD_600__nm_) at time 0, 1, 3, 6, 9, 12, 24, and 48 h. Optical densities were examined at OD_600__nm_ on a Smartspec Plus Spectrophotometer (Bio-Rad Laboratories Inc.) and colony forming unit (CFU) counts were performed at 6, 24, and 48 h on MRS or MRS-ERM agar plates to examine growth.

### Generation of Bacterial Supernatants

To generate bacterial Conditioned media (CM) for cell treatment, *L. reuteri* 6475, *L. reuteri* 6475:*folC2*, and *L. reuteri* 6475:*folC* were grown at exponential phase in MRS and used to inoculate LDM4 (OD_600__nm_ adjusted to 0.1). Cultures were grown anaerobically at 37°C for 24 h (stationary phase). Following incubation, cells were pelleted by centrifugation (5,000 × *g* for 5 min) and the supernatant was treated with DTT (Sigma 10708984001) to release protein-bound folates, then filtered using Amicon Ultra-15 centrifugal filter units using the ultracel-3 membrane (Millipore UFC900324, Millipore, Bedford, MA, United States). The filtrate (<3 kDa) was lyophilized and resuspended in enteroid media to generate 25% CM in the appropriate cell culture medium. All CM was pH-adjusted to pH = 7.4.

### Folate Quantification

Bacterial cultures were collected at exponential or stationary phases (strain-specific media and incubation duration in Supplementary Table S1). Cultures were centrifuged at 3,000 × *g* for 15 min at 4°C. Bacterial culture supernatants were reserved for folate quantification. The cell pellet was resuspended in STE buffer (100 mM NaCl, 10 mM Tris-HCl, pH 8.0, 1 mM Ethylenediaminetetraacetic acid (EDTA), pH 8.0) and centrifuged at 7,500 × *g* for 5 min at 4°C, and supernatants were removed. Bacterial cell pellets were resuspended in 100 μl of “Fol diluent,” included with the ARCHITECT Folate Assay (Abbott Laboratories, Abbott Park, IL, United States). One hundred microliters of culture supernatants was diluted in 100 μl of “Fol diluent.” The concentration of folate present in each sample was determined using the ARCHITECT Folate assay, a competitive immunoassay using direct chemiluminescence. Samples were treated with 0.1 M DTT for 5 min to release folate conjugates from binding proteins in the sample. Folate quantification was determined through a competitive binding assay utilizing an acridinium–folate-bound biotin–folate-binding protein. Binding was detected by the ARCHITECT *i* System using avidin-conjugated paramagnetic solid phase particles. The inter- and intra-assay variation of the assay was <10% and recovery of added folate was >90%.

### MALDI TOF MS Analysis of Bacterial Folates

Bacterial cultures were collected at exponential or stationary phases (strain-specific media and incubation duration in Supplementary Table S1). The cultures were centrifuged at 4,500 × *g* for 10 min at 4°C, and bacterial supernatants were removed. Bacterial cell pellets were washed with water and centrifuged at 6,000 × *g* for 10 min at room temperature. Folates were extracted from cell pellets by adding extraction buffer (0.1% TFA, 50% acetonitrile in water), vortexing, and centrifuging at 16,000 × *g* for 10 min at room temperature. The folate-containing supernatants were spotted in CHCA matrix (ProteoChem P9100, Hurricane, UT, United States) onto a MALDI target (SimuTOF Systems, Marlborough, MA, United States). A standard consisting of eight peptides ranging from 8 to 28 amino acids was spotted onto the MALDI target as calibrant. Spectra were acquired on a SimulTOF Combo 200 Mass Spectrometer (Virgin Instruments, Marlborough, MA, United States) in reflector mode and annotated with MoverZ software (Proteometrics, LLC, New York, NY, United States).

### Human Gastrointestinal Microbial Genome Analysis

Distribution of the folate synthesis genes in the gastrointestinal reference genomes of the Human Microbiome Project (HMP) was determined using the Integrated Microbial Genomes and Metagenomes (IMG/M) (Markowitz et al., 2012) available through the Joint Genomes Institute (JGI). Version 5.0 (updated August of 2018) was accessed through https://img.jgi.doe.gov/ on November 14, 2018. The Profile and Alignment Tool (in JGI) was used to determine identities of folate biosynthesis genes present in the 512 gastrointestinal bacterial reference genomes of the HMP. Genes encoding enzymes involved in folate synthesis were designated by the enzyme commission (E.C.) number. The E.C. number for each enzyme involved in folate synthesis was queried against each genome using the Profile and Alignment Tool in JGI. Folate metabolic capacity was established on the basis of genes present in each genome.

### Evaluation of Bacterial RNA by qPCR

Bacterial cells were fixed by adding an equal volume of cold methanol to exponential or stationary phase bacterial cultures. Fixed cells were pelleted at 16,000 × *g* for 1 min. RNA was isolated from bacterial cell pellets using the QuickRNA kit (Zymo Research, Irvine, CA, United States) according to the manufacturer’s instructions with modifications. Briefly, bacterial pellets were resuspended in 100 μl STE buffer (100 mM NaCl, 10 mM Tris–HCl, pH 8.0, 1 mM EDTA), and transferred to tubes containing 0.1 mm glass beads. Samples were disrupted on a FastPrep bead homogenizer (MP Biologicals) for 20 s at 4.0 m/s. Following disruption, Zymo Quick RNA lysis buffer was added to each glass bead containing tube, and the samples were disrupted on the FastPrep homogenizer for a second time. Cell debris and beads were removed by centrifugation at 10,000 × *g* for 1 min, and the supernatant was processed for RNA according to the manufacturer’s protocol. Genomic DNA removal was performed using the Ambion Turbo DNA-free kit following manufacturer instructions (ThermoFisher, Waltham, MA, United States). Quantitation and quality control (A260/A280 and 260/230 ratios) were performed with a Nanodrop OneC (ThermoFisher Scientific, Waltham, MA, United States) Spectrophotometer. Synthesis of cDNA from RNA was completed by reverse transcription using the SensiFast cDNA synthesis kit (Bioline, London, United Kingdom). For quantitative real-time PCR (qRT-PCR) analysis of bacterial folate genes, bacterial cDNA was examined using FAST SYBR green and primers (Supplementary Table S4) on a Stratagene 3005p qPCR System (ThermoFisher, Waltham, MA, United States). For relative quantitation, the ΔΔCt method was used and microbial mRNA quantities were normalized to amounts of 16S rRNA.

### Treatment of Human Colonoid Monolayers With Bacterial Folates

Differentiated human colonoid monolayers were generated as previously described (Zou et al., 2017; Rajan et al., 2018). The colonoid lines AsC109 and C103 were established from colonic tissue sampled via endoscopic-guided biopsy procedure from the ascending colon (Baylor College of Medicine, IRB # H-35094). Three-dimensional colonoids were cultured in complete medium with growth factors (CMGF+) as previously described (Sato et al., 2011; Saxena et al., 2016). Briefly, CMGF+ consisted of advanced Dulbecco’s modified Eagle medium (DMEM)/F-12 medium (Invitrogen) supplemented with 1× GlutaMAX (Invitrogen), 10 mM HEPES buffer (Invitrogen), and 100 U/mL penicillin–streptomycin (Invitrogen), with 50% (v/v) Wnt3A-conditioned medium, 20% (v/v) R-spondin conditioned medium, 10% (v/v) Noggin-conditioned medium, 1× N-2 supplement (Invitrogen), 1× B-27 supplement (Invitrogen), 1 mM *N*-acetylcysteine (Sigma–Aldrich), 50 ng/mL mouse epidermal growth factor (EGF) (Invitrogen), 10 nM Leu-Gastrin I (Sigma–Aldrich), 500 nM A-83-01 (Tocris Bioscience), 10 nM SB202190 (Sigma–Aldrich), and 10 mM nicotinamide (Sigma–Aldrich, St. Louis, MO, United States). Colonoids were passaged in phenol red-free, growth factor-reduced Matrigel (Corning).

Colonoids at passage 6 were seeded into flat 96-well plates as described previously (VanDussen et al., 2015; Ettayebi et al., 2016) to generate monolayers. Briefly, 96-well plates were pre-treated with Matrigel diluted in 1× phosphate-buffered saline (PBS) (1:40) and incubated at 37°C. The Matrigel was removed from 3D colonoids and the colonoids were washed with an ice-cold solution of 0.5 mM EDTA in 1× PBS and dissociated with 0.05% trypsin/0.5 mM EDTA at 37°C for 4 min. Following the incubation, the trypsin was inactivated with advanced DMEM/F12, 1× Glutamax, 1× HEPES + 10% fetal bovine serum. The cell solution was pipetted vigorously and filtered via a 40 μm nylon cell strainer (Falcon, Cat. No. 352340). The resulting single cells were centrifuged at 400 × *g* for 5 min, resuspended with CMGF+ and 10 μM Y-27632 Rock inhibitor, and plated into Matrigel-coated wells. After 48 h, the medium was changed to differentiation medium, which contains the same components as CMGF+ without Wnt3A conditioned medium, R-spondin conditioned medium, SB202190, and nicotinamide and only 5% (v/v) Noggin conditioned medium, with the addition of 10 μM Y-27632 Rock inhibitor. Differentiation medium with Y-27632 was changed daily for 4–5 days to differentiate cells.

For bacterial supernatant treatment, the differentiation media on colonoid monolayers on 96-well plates was changed to DMEM without folate supplemented with 1× HEPES, 1× Glutamax, and 1× pyruvate. Colonoids were treated for 16 h with either media alone, 25% CM from uninoculated LDM4, wild-type *L. reuteri* 6475, *L. reuteri* 6475:*folC* (gene required for pteroate glutamylation), or *L. reuteri* 6475:*folC2* (gene required for THF synthesis) (Thomas et al., 2016) in the minimal medium. Following treatment, 60 μM resazurin was added to the media and metabolic activity was examined by a change in fluorescence (excitation 560 nm: emission: 600 nm) after 2 h of incubation. Next the monolayers were gently dissociated with PBS (Ca^2+^, Mg^2+^ free) containing 3 mM EDTA, 10 mM glucose, and 10 μl cell solution was mixed with 10 μl trypan blue to assess viability. Cell counts were determined using a Countess Automated Cell Counter (Invitrogen, Carlsbad, CA, United States) [*n* = 2 biological replicates (enteroid lines), three technical replicates].

### Mammalian RNA Extraction and RNAseq

To assess the transcriptional profile of human intestinal enteroids, sterile monolayers were prepared on transwells and differentiated as previously described (Zou et al., 2017). RNA was extracted via TRIZOL according to the manufacturer’s instructions and rRNA integrity was checked on a 2100 Bioanalyzer (Agilent, Santa Clara, CA, United States) from two independent biological replicates per segment. Using mRNA enriched samples by Novogene, paired-end Illumina sequencing was performed following the standard work-flow procedure. Raw sequence reads were mapped to human genome hg19 using STAR software to generate the number of fragments per kilobase per million mapped reads (FPKM) for each gene (*n* = 2 biological replicates).

### Evaluation of Mammalian RNA by qPCR

Colonoid RNA was extracted via TRIZOL according to the manufacturer’s instructions and genomic DNA removal was performed using the Ambion Turbo DNA-free kit (ThermoFisher, Waltham, MA, United States). Quantitation and quality control (A260/A280 and 260/230 ratios) were performed with a Nanodrop OneC (ThermoFisher Scientific, Waltham, MA, United States) Spectrophotometer. cDNA was synthesized from RNA by reverse transcription using the SensiFast cDNA synthesis kit (Bioline, London, United Kingdom). Expression of key folate-related genes was analyzed using qRT-PCR. The reaction was performed with colonoid cDNA and FAST SYBR green master mix (Applied Biosystems, Foster City, CA, United States) per manufacturer instructions. The following amplification conditions were used: after an initial denaturation at 95°C for 20 s, 40 amplification cycles were performed at 95°C for 3 s and 60°C for 30 s on a Stratagene 3005p qPCR System (ThermoFisher, Waltham, MA, United States). Primers used in this study are listed in Supplementary Table S4. The ΔΔCt method was used for relative quantification and mRNA levels were normalized to human 18S.

### Statistical Analyses

Statistical analyses were performed using GraphPad Prism version 198 5.04 (GraphPad Software, San Diego, CA, United States). All data were normally distributed and examined using non-parametric tests. All data were analyzed by one-way ANOVA with a Bonferroni *post hoc* test. Statistical test results are presented as mean ± standard deviation (SD). *P*-values are indicated by asterisks as follows: ^∗^*p* < 0.05.

## Results

### Folate-Generating Capacity Is Ubiquitous Throughout the Human Intestinal Microbiome

**FIGURE 1:**
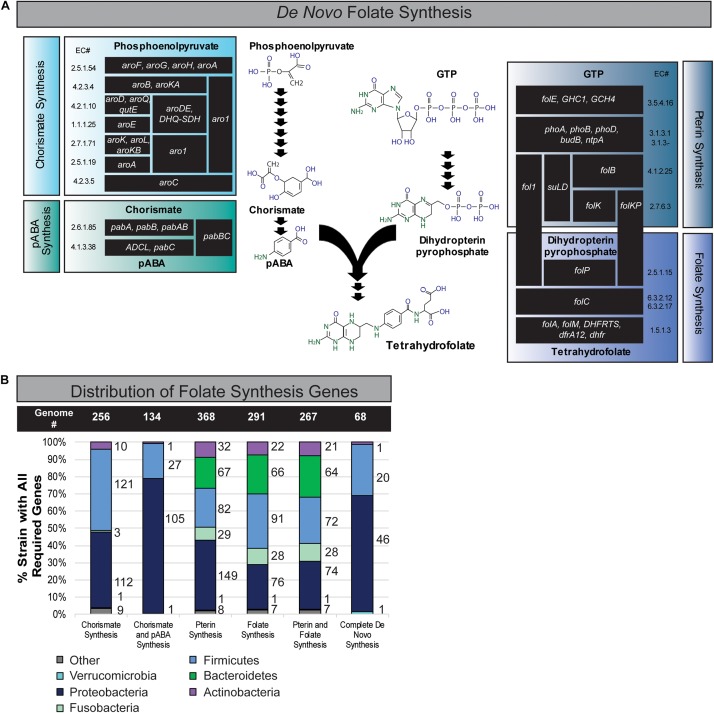
Distribution of folate synthesis genes in the gastrointestinal human microbiome. **(A)** Schematic of *de novo* folate synthesis divided into four modules: (1) chorismate synthesis, (2) pABA synthesis, (3) pterin synthesis, and (4) folate synthesis. Each protein is listed by name and enzyme commission (E.C.) number. **(B)** Distribution of genomic folate synthesis capabilities within the Human Microbiome Project’s Gastrointestinal Reference Genomes stratified by phylum.

The folate metabolic capacity of 512 well-defined, phylogenetically diverse reference bacterial genomes from the human gastrointestinal tract was evaluated. Bacterial genomes were binned as potential contributors of folate biosynthesis if the genome contained at least one set of genes representing folate biosynthetic metabolic pathway. Pathways include genes required for synthesis of (1) chorismate from phosphoenolpyruvate (seven enzymatic functions), (2) pABA from chorismate (two enzymatic functions), (3) dihydropterin pyrophosphate from GTP (four enzymatic functions), and (4) THF from pABA and dihydropterin pyrophosphate intermediates (four enzymatic functions) (Figure 1A). A summary of the relative capacity of these gut microbes to synthesize specific folate intermediates is illustrated in a stacked graph stratified by phylum (Figure 1B).

Chorismate acts as an intermediate for diverse metabolic pathways including folate and amino acid biosynthesis and the genes required to synthesize chorismate from the glycolysis product, phosphoenolpyruvate, were found in 256 genomes (Figure 1B). These genomes represented all key phyla except Bacteroidetes. We found 134 genomes encode the capacity to convert chorismate to pABA including microbes from the phyla Proteobacteria, Firmicutes, Actinobacteria, and Fusobacteria. A total of 368 genomes, 72% of the reference genomes, representing six key intestinal phyla encode the capacity of synthesizing dihydropterin pyrophosphate from GTP (Pterin Synthesis). Genes involved in the synthesis of THF from pABA and dihydropterin pyrophosphate (folate synthesis) were found in 291 (57%) of the bacterial genomes representing six key phyla of the human intestinal microbiome, Firmicutes, Bacteroidetes, Actinobacteria, Fusobacteria, Proteobacteria, and Verrucomicrobia (Figure 1B). A total of 392 (77%) bacterial genomes contain genes capable of synthesizing THF if pABA is present as a precursor (Figure 1B). We also found that 267 genomes had the capacity for both pterin and folate synthesis. Finally, we found that 68 genomes were capable of using chorismate to synthesize folates completely *de novo* (Figure 1B). The phyla Firmicutes and Proteobacteria exhibited the highest number of genomes capable of *de novo* folate synthesis. This dataset indicates that the majority of microbes must obtain precursors in the form of dietary nutrients or microbial cross-feeding in order to produce luminal folate in the intestine.

### Functional Characterization of Folate Production by Intestinal Microbes

**FIGURE 2:**
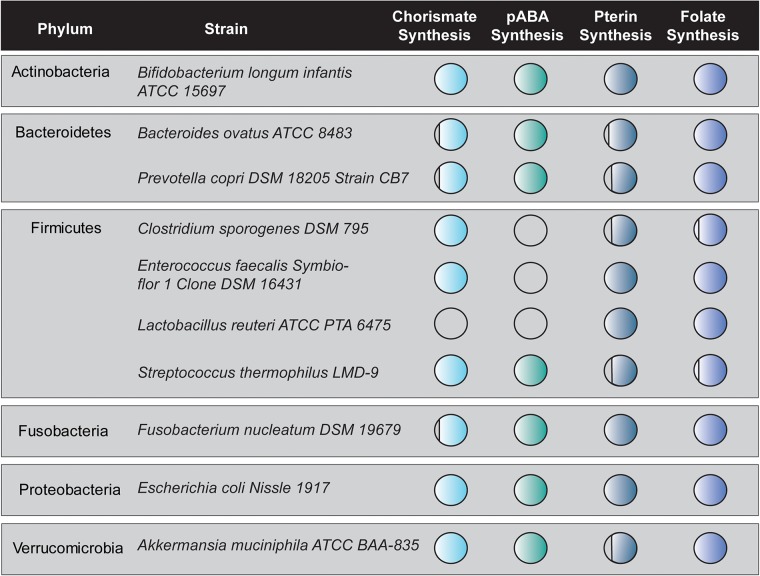
*In silico* analysis of folate synthesis gene distribution in an intestinal microbe cohort. The Joint Genomes Institute (JGI) Profile and Alignment Tool was used to predict chorismate synthesis genes, pABA synthesis genes, pterin synthesis genes, and folate synthesis genes in a cohort of 10 strains representing six phyla of the human intestinal microbiome. The circles are filled in proportion to the number of those genes contained in the genome of each bacterial strain. Circles 90% filled represent one missing gene while circle 75% filled represent two missing genes.

The biochemical functionality of bacterial folate biosynthesis genes identified *in situ* was verified in a cohort of 10 bacterial strains representing six key phyla of the human intestinal microbiome. These microbes included three strains which are currently available as probiotics (*L. reuteri* ATCC PTA 6475, *Enterococcus faecalis* Symbioflor DSM 16431, and *E. coli* Nissle 1917) (Gronbach et al., 2010; Mu et al., 2018; Baccouri et al., 2019). Figure 2 depicts the genetic capacity of 10 bacterial strains to synthesize folate *de novo*. Circle shading corresponds to genetic capacity; with completely shaded circles representing complete gene sets for the indicated pathways and empty circle indicating no genes present (Figure 2). From this representative cohort, only two strains (*B. longum* subsp. *infantis* ATCC 15697 and *E. coli* Nissle 1917) representing two phyla (Actinobacteria and Proteobacteria) contain complete pathways for generating folate *de novo*. In the phylum Firmicutes, three of four strains (*C. sporogenes* DSM 795, *E. faecalis* Symbioflor DSM 16431, and *L. reuteri* ATCC PTA 6475) are completely devoid of genes necessary to synthesize pABA and hence their ability to synthesize folate depends on the presence of pABA in their environmental milieu. This is consistent with previous work demonstrating the requirement of pABA for *L. reuteri* folate-production (Spinler et al., 2014; Thomas et al., 2016).

To verify our *in situ* findings, we characterized folate production by one strain representing each of the six major intestinal phyla: *B. longum* subsp. *infantis*, *C. sporogenes*, *L. reuteri*, *E. coli* Nissle, *A. muciniphila*, and *P. copri*. We cultured each strain to exponential and stationary phase growth since bacterial metabolism differs significantly between growth phases (Rolfe et al., 2012; Robador et al., 2018; Supplementary Table S1). We collected supernatants and cell pellets and analyzed folate concentrations by competitive immunoassay. The intermediate pABA was included in all defined medias (LDM4 and CDMM). We found that in exponential growth phase, *B. longum* subsp. *infantis* (123.3 ± 11.2 ng/ml), *L. reuteri* (62.6 ± 1.8 ng/ml), and *A. muciniphila* (64.4 ± 0.15 ng/ml) produced net amounts of folate, while *C. sporogenes*, *E. coli* Nissle, and *P. copri* were folate consumers (Table 1). However, in stationary phase the majority of bacteria were net folate producers, with *B. longum infantis* generating the highest concentration (261.1 ± 0.15 ng/ml). Based on net production or consumption of folate, representative intestinal microbes can be divided into three functional groups: folate consumers, folate producers, and conditional producer/consumer. Regardless of growth phase, *P. copri* DSM 18205 is a net folate consumer and *B. longum* subsp. *infantis* ATCC 15697 is a net folate producer. *E. coli* Nissle 1917, on the other hand, is a conditional producer, consuming folate in exponential phase and producing folate during stationary phase (Table 1). This *in vitro* data indicate that several intestinal species are capable of generating folate.

**TABLE 1:** Total folate production quantified by chemiluminescence immunoassay.

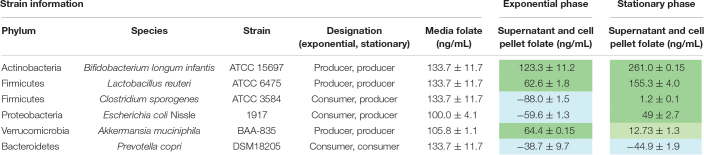

*All data are represented as average ± *SD* (*n* = 3 biological replicates). Total bacterial folate (ng/mL) was determined by subtracting the amount of folate in the uninoculated media from the sum of the average folate concentration in the bacterial supernatant and the average folate concentration in the cell pellet. The colors indicate production of folate (green) or consumption of folate (blue).*

### Different Folylpolyglutamate Profiles Generated by Intestinal Microbes

**FIGURE 3:**
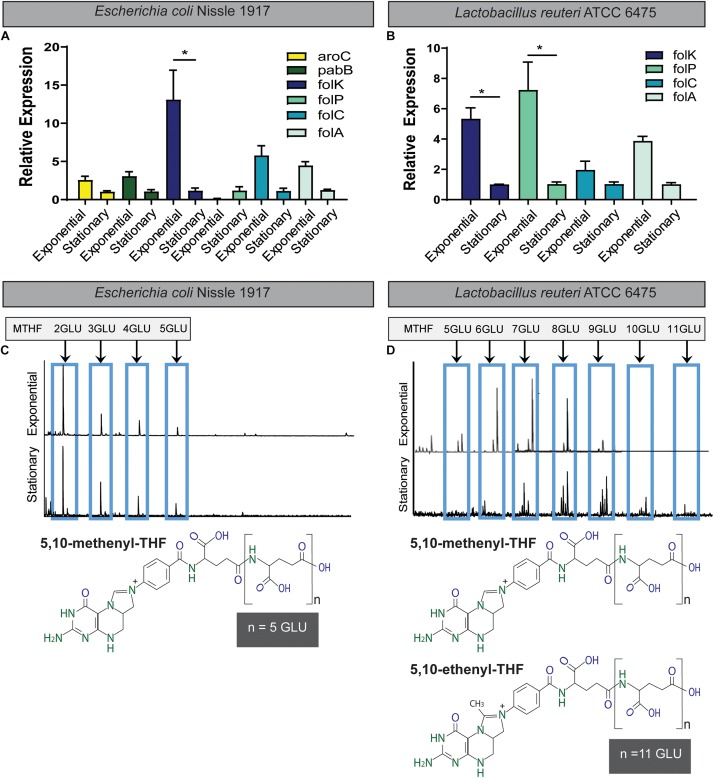
Folate synthesis during exponential and stationary growth phase by qPCR and MALDI-TOF mass spectrometry. Expression of folate synthesis genes at exponential and stationary phase growth in **(A)** *Escherichia coli* Nissle 1917 and **(B)** *Lactobacillus reuteri* ATCC PTA 6475 as determined by qPCR. *aroC* is involved in chorismate synthesis; *pabB* in pABA synthesis; *folP*, *folC*, and *folA* in pterin and folate synthesis. Expression is normalized to 16S rRNA. One-way ANOVA with Bonferroni *post hoc* test. ^∗^*p* < 0.05 (*n* = 3 biological replicates). Differential glutamylation profiles of folates determined by MALDI-TOF mass spectrometry for **(C)** *E. coli* Nissle 1917 and **(D)** *L. reuteri* ATCC PTA 6475. *L. reuteri* produces both the 5,10-methenyl THF as well as the 5,10-ethenyl THF form with + 14 glutamate (GLU) residues. Blue boxes highlight the peaks which represents MTHF + *n*GLU.

We examined two members of the gut microbiota, *E. coli* and *L. reuteri*, to assess expression of folate genes and the ability to polyglutamylate folate. Consistent with folate production (Table 1), qPCR analysis of key folate genes indicated increased expression in the exponential phase for *E. coli* Nissle 1917 (Figure 3A). Genes such as *folK*, the final enzyme required for pterin synthesis, were upregulated in *E. coli* Nissle 1917 exponential phase compared to stationary phase. Similar to *E. coli* Nissle 1917, *folK* was also upregulated in *L. reuteri* ATCC 6475 during exponential phase. Additionally *folP*, the enzyme which combines the pterin and pABA intermediates was significantly upregulated in exponential phase in *L. reuteri* ATCC 6475. Next, we examined glutamylation profiles produced by these strains. The folates synthesized by *E. coli* Nissle 1917 contain relatively constant glutamate tails (3–5 glutamate residues) and do not lengthen with increased time of incubation (Figure 3C). In contrast, folates synthesized by *L. reuteri* 6475 produce relatively long glutamate tails (FPGs; chain length 7–9 glutamate residues), which increase in length by adding glutamate residues over time (Figure 3D). In addition, to the simple 5,10-methenyl THF (tetrahydrofolic acid) polyglutamate polymers produced by *E. coli* Nissle 1917, *L. reuteri* 6475 produces both 5,10-methenyl THF polyglutamates and a unique 5,10−ethenyl THF with a covalently linked γ-polyglutamate tail (Roth et al., 2019). 5,10-Ethenyl THF carries two carbons at the “business end” of THF, between N5 and N10. It contributes to a two-carbon variant of the folate cycle that leads to the production of ethionine instead of methionine (Roth et al., 2019). These data are among the first to identify commensal gut microbes with unique patterns of glutamylated folate, which may have an impact on host folate processes.

### Bacterial Folate-Mediated Modulation of Human Folate Transporter Gene Expression

**FIGURE 4:**
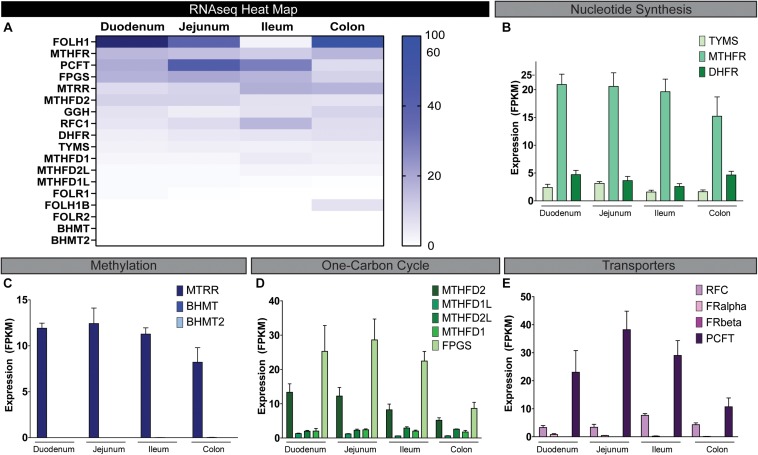
RNAseq expression of folate-associated genes in human enteroids. Enteroids derived from diverse intestinal segments were examined by RNAseq. **(A)** Heat map of the expression of folate-associated genes in duplicate biological samples from human duodenum, jejunum, ileum, and colon enteroids. Expression is indicated from high (dark blue) to low (white). The data were divided into four groups based on gene expression: nucleotide synthesis **(B)**, methylation **(C)**, one-carbon cycle **(D)**, and folate transporters **(E)** (*n* = 2 biological replicates).

The novel human enteroid model has transformed the study of intestinal epithelial cells. Enteroids derived from intestinal stem cells represent non-transformed, multicellular epithelial cultures that can express all the native differentiated intestinal cell types (Sato et al., 2009, 2011; Clevers, 2016; Saxena et al., 2016; Zou et al., 2017). Enteroids appear to recapitulate human intestinal physiology, making them an ideal model to examine folate-associated processes (Foulke-Abel et al., 2016; Saxena et al., 2016; Zou et al., 2017). Moreover, human enteroids grown as 2D monolayers represent an optimal tool for examining host–microbe interactions. Using RNAseq, we identified several folate-related genes in human enteroids derived from duodenum, jejunum, ileum, and colon (Figures 4A–E). High expression was observed in genes associated with nucleotide synthesis, methylation, one-carbon cycle, and folate transporters (Figures 4B–E). The most highly expressed genes included methylenetetrahydrofolate reductase (MTHFR), folylpolyglutamate synthase (FPGS), 5-methyltetrahydrofolate-homocysteine methyltransferase reductase (MTRR), PCFT (SLC46A1) and methylenetetrahydrofolate dehydrogenase (NADP + Dependent) 2 (MTHFD2), methenyltetrahydrofolate cyclohydrolase, and folate hydrolysis (FOLH) (Figure 4A). These genes represent unique host pathways involved in folate homeostasis. Interestingly, of these highly expressed genes, robust expression was observed in the small intestine (duodenum, jejunum, and ileum) and to a slightly lesser degree in the colon. FOLH is responsible for converting FPG to mono-glutamate which can then be absorbed by mammalian cells and FOLH1 expression was high throughout all intestinal segments, including the colon (35.7 ± 12.5 FPKM). Since the colon is the primary site of microbe-produced folate absorption (Pompei et al., 2007a; Aufreiter et al., 2009; Lakoff et al., 2014), we chose to focus our attention on the colon.

We modeled host–microbe interactions by examining folate receptor/transporter gene expression in response to folate generated by *L. reuteri* 6475. We also included two *L. reuteri* 6475 mutants which harbored insertions in either the *folC* gene (*6475*:*folC*) or the *folC2* gene (*6475:folC2*). Our previous study showed that 6475:*folC* lacks the ability to produce a polyglutmate tail on 5,10-methenyl THF and that *6475:folC2* does not produce any 5,10-methenyl THF (Thomas et al., 2016). We confirmed that these mutants exhibit normal growth in LDM4 by optical density (OD_600__nm_) and CFU over 48 h (Supplementary Figures S1A,B). The CFU counts of all *L. reuteri* strains (WT, *6475:folC*, or *6475:folC2*) after 24 h of growth mirror the concentrations of *L. reuteri* observed in patients (10^8^) (Million et al., 2013) and concentrations that are well tolerated and safe in humans (Mu et al., 2018). As a result, 24 h was selected for further analysis. By mass spectrometry, we quantified the folate produced by our *L. reuteri* strains after 24 h growth (Supplementary Figure S1C). Consistent with our previous work, we demonstrate that the *6475:folC* mutant is capable of producing glutamylated folate (170 ± 25.6 ng/ml), while the *6475:folC2* mutant is unable to produce folate above the LDM4 media control (16.7 ± 1.2 ng/ml) (Supplementary Figures S1D,E).

**FIGURE 5:**
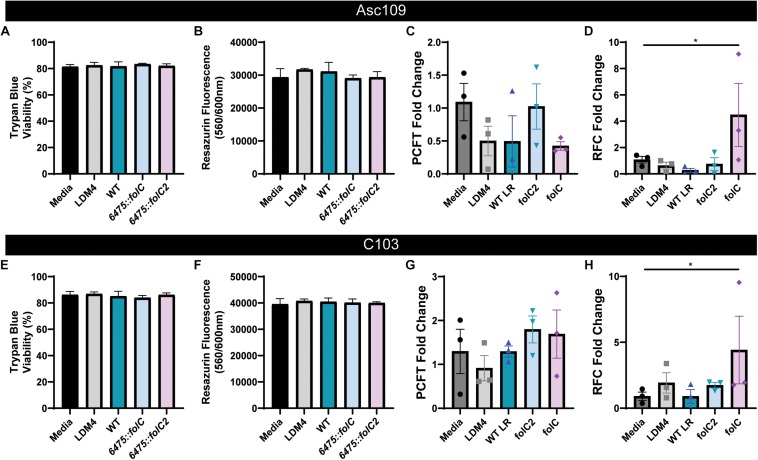
Expression of folate transporters in human colonoids treated with *L. reuteri* conditioned media. Human colon monolayers (lines Asc109 and C103) were incubated with 25% uninoculated bacterial medium (LDM4) or 25% *L. reuteri* ATCC 6475 conditioned media for 16 h. In addition to WT *L. reuteri*, the 6475:folC mutant, which can produce THF, but can’t synthesize a polyglutamate tail, and the 6475:folC2 mutant, which can’t synthesis any THF, were included in the analysis. Viability assessment of colonoids following 16 h incubation as determined by **(A,E)** trypan blue staining and **(B,F)** resazurin assay. Expression of proton-coupled folate transporter, PCFT **(C,G)** and reduced folate carrier, RFC **(D,H)** determined by qPCR normalized to 18S. One-way ANOVA with Bonferroni *post hoc* test. ^∗^*p* < 0.05 (*n* = 2 biological replicates, three technical replicates).

**FIGURE 6:**
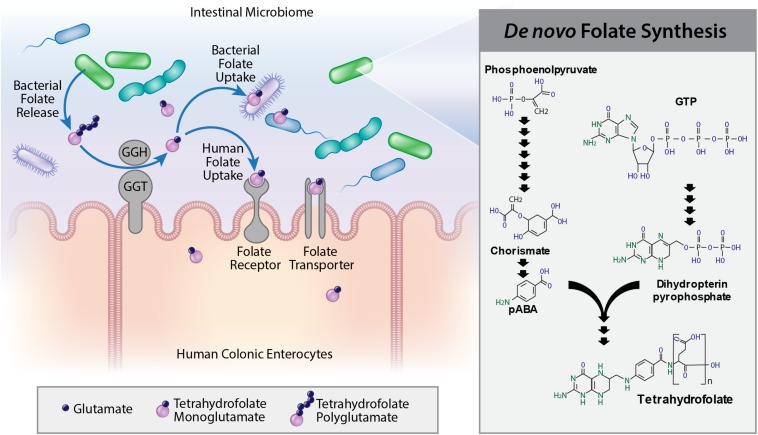
Model of microbial folate production and microbe:host interactions. Polyglutamylated tetrahydrofolates are synthesized by the human intestinal microbiome. Human gamma-glutamylhydrolase, GGH, and gamma-glutamyltransferase, GGT, remove glutamate moieties making monoglutamylated folates available for uptake by other luminal bacteria or human colonocytes. Folate can activate folate receptors (Frα or Frβ) or be taken up into enterocytes by folate transporters (RFC or PCFT).

Using wild-type *L. reuteri* 6475 and insertion mutants *6475:folC* and *6475:folC2* as a model for microbe folate production, the relative abilities of microbial-derived folates to modulate human folate receptor gene expression could be examined. Two human colonoid lines were used for all analyses: Asc109 and C103. Colonoid monolayers were treated with DMEM containing no folate in the presence of 25% LDM4 or 25% *L. reuteri* (WT, *6475:folC*, or *6475:folC2*) LDM4 CM overnight (16 h). After treatment, the colonoid monolayers maintained their intestinal architecture as observed by light microscopy (data not shown). To confirm the viability of the enteroid monolayers following treatment, we assessed viability by trypan blue staining (Figures 5A,E) and metabolic activity by resazurin assay (Figures 5B,F). Consistent with the microscopic images, we observed no changes in viability or metabolic activity in colonoids treated with either *L. reuteri* strain. Analysis of mRNA levels of the PCFT (Figures 5C,G) and reduced folate transporter (RFC) (Figures 5D,H) by qPCR revealed unique patterns in response to our bacterial supernatant. Application of bacterial supernatants had no effect on the levels of the PCFT in either colonoid line (Figures 5C,G). However, the application of bacterial supernatants containing a non-glutamylated folate (*L. reuteri 6475:folC*-mutant strain) to human colonoids yielded enhanced expression of the folate transporter RFC (Figures 5D,H). This effect was not observed with the *L. reuteri 6475:folC2*-mutant strain which is unable to synthesize folate, pointing to the potential role of non-glutamylated folate in modulating RFC levels. In addition to receptor levels, other folate-related genes were queried (Supplementary Figure S2). No changes were observed in the GCPII (folh1), which is required to deconjugate polyglutamylated folates, with any of the treatment conditions (Supplementary Figures S2A,B). Likewise, no significant changes were observed in any MTHFD genes (Supplementary Figures S2C–H) or serine hydroxymethyltransferase 2 (SMTH2) (data not shown). Together these data demonstrated that bacteria in the human gastrointestinal microbiome produce bioavailable folates and that the glutamylation status of microbial folate can influence folate transporter expression in the human intestine (Figure 6).

## Discussion

Our study has demonstrated that folate biosynthesis is a major biochemical feature of the human intestinal microbiome. Four basic metabolic modules in folate biosynthesis have been defined, and described in terms of prevalence of these separate modules in the reference set of 512 microbial genomes attributed to the human gastrointestinal tract. These modules include chorismate, pABA, pterin, and folate synthesis. Our data demonstrate that these genes are distributed among the six phyla of the human microbiome (Actinobacteria, Bacteroidetes, Firmicutes, Proteobacteria, Fusobacteria, and Verrucomicrobia). However, only strains from Proteobacteria and to a lesser degree Firmicutes, Actinobacteria, and Verrucomicrobia (68 strains in total) are able to completely generate folate *de novo*. This study provides insight into the genetics of the human intestinal microbiome and suggests a cooperative role of microbial communities to generate B complex vitamins in the human host. Our studies revealed different glutamylation profiles for commensal gut microbes that may affect folate bioavailability in the intestine. We specifically showed that *L. reuteri* and *E. coli* Nissle generate moderate amounts of folate (49–77 ng/μl) and differ in the extent of polyglutamylation. Finally, our data showed that in the absence of bacterial THFs, folate transporter expression in human colonic enterocytes is significantly increased. This work has advanced the scientific understanding of bacterial folate metabolism and expanded the potential role for the microbiome in treating vitamin deficiencies.

Folate deficiency contributes to serious health problems and can result from insufficient dietary intake, poor absorption of ingested folate, and altered folate metabolism due to genetic defects or drug interactions (Rajagopalan et al., 2002; Hiraoka and Kagawa, 2017). In the absence of a genetic inability to absorb folate, folate deficiency can be treated through dietary changes, increasing consumption of dark green vegetables, beans, liver, and folate-enriched foods. Long-term correction of low folate through the diet has yielded mixed results (Menon et al., 2005; Kemperman et al., 2006; Nair and Iyengar, 2009; Kaehler et al., 2012). Folate deficiency can also be treated by supplementation with folic acid, a synthetic, fully oxidized, mono-glutamylated form of folate. In contrast to the synthetic folic acid, naturally occurring dietary and microbe-produced folates are polyglutamylated, fully reduced THFs with methyl, methylene, or formyl groups. THF is the form of folate that can be processed through the one-carbon cycle, making this form a more desirable format for absorption. As a result, we speculate that the delivery of microbial folates may be amenable for the treatment of low folate. Microbe-derived folate is particularly attractive for patients with inactivating single-nucleotide polymorphisms (SNPs) in dihydrofolate reductase (DHFR), which is required to convert folic acid to THF. These patients are unable to efficiently absorb synthetic folates (Askari and Krajinovic, 2010; Hiraoka and Kagawa, 2017) and thus need alternative sources of THFs. By delineating the mechanisms regulating the production and secretion of folate by the microbiome, manufacturers, physicians, and researchers can make informed decisions regarding probiotic recommendations and nutritional counseling based on human microbiome science.

We found that 13.3% of 512 bacterial reference genomes from the human intestinal microbiome contain the genes spanning all four metabolic modules involved in *de novo* synthesis of THF. This sizeable fraction of intestinal microbes containing complete sets of folate biosynthetic genes suggests that the microbiome serves as an important folate-generating “organ.” The remaining genomes (>86%) require folate or folate intermediates from other bacteria of the human microbiome or the human diet. Therefore, when proposing a potential probiotic source of folate, either complete capacity to generate folates or effective combinations of folate producing strains (combining a pABA-producing strain with a pABA-dependent strain) should be considered. Our data are consistent with previous genomic studies that predicted that 26% of Actinobacteria, 79% of Fusobacteria, 71% of Proteobacteria, and 15% of Firmicutes in the human intestinal microbiome are fully capable of synthesizing folate *de novo* (Magnusdottir et al., 2015). Magnusdottir et al. (2015) suggested that the human microbiome was potentially capable of synthesizing 37% of the daily recommended intake of folate for non-pregnant, non-breastfeeding adults. This recommendation highlights the potential importance of the gut microbiome as a source of folate production. We have identified strains including *B. ovatus* ATCC 8483 and *A. muciniphila* ATCC BAA 835 that contain the complete complement of 16 enzyme functions needed to synthesize folates. These strains have not historically been considered probiotics and as a result remain uncharacterized and may have undesired effects. Therefore, it is advantageous to utilize a well-characterized probiotic strains for folate delivery. The human breast milk-derived strain *L. reuteri* ATCC PTA 6475 has been used in clinical trials (NCT02422082, NCT00774163, NCT03360253) and is known to be safe to animals and humans (Adams and Marteau, 1995; McCabe et al., 2013; Britton et al., 2014; Fatheree et al., 2017; Galofre et al., 2018; McCabe and Parameswaran, 2018; Schepper et al., 2019). The genome of *L. reu*teri ATCC PTA 6475 lacks genes needed to synthesize chorismate or pABA, and depends on folate precursors. As a result, we propose addition of *L. reuteri* and pABA to harness the full folate producing potential of *L. reuteri*.

Knowledge of folate-related genes in the intestine has been limited by the lack of clinically relevant systems. The majority of existing work has been performed in rodent models and cancer-derived cell lines. Work using PCFT-deficient mice has identified that the presence of PCFT is a requirement for the transport of folates across the apical brush-border membrane of the proximal small intestine (Wang et al., 2013; Visentin et al., 2014). In immortalized rat small intestinal epithelial IEC-6 cells, PCFT was also found to facilitate entry of folate at low pH, while RFC enabled folate absorption at neutral pH (Rajgopal et al., 2001). Both these studies focused on the small intestine and did not examine the colon, which is the primary site for microbe-produced folate absorption (Pompei et al., 2007a; Aufreiter et al., 2009; Lakoff et al., 2014). Although PCFT is present in the colonic mucosa, the extent of colonic folate absorption *in vivo* is not known. Rodents are the experimental tool of choice for the majority of intestinal absorption studies and these types of studies have yielded tremendous insight into intestinal homeostasis. Nevertheless, significant differences between mice and humans may affect our knowledge of folate transport (Mestas and Hughes, 2004). Lakoff et al. (2014) verified that humans can absorb folates through the colon using a pH-sensitive capsule for targeted delivery. However, this study did not evaluate the composition of the human microbiome in their subjects or the cumulative effects of microbial metabolites on folate absorption as it used a single defined monoglutamylated folate. Our work is the first to identify folate-related processes in epithelial cells within different intestinal regions based on human intestinal enteroids. Human intestinal enteroids yield a model of intestinal epithelium exhibiting a similar cellular composition and function as the intact epithelium of the human intestine (Sato et al., 2011; Sato and Clevers, 2013; Foulke-Abel et al., 2014, 2016; Middendorp et al., 2014; Saxena et al., 2016; Zachos et al., 2016). Additionally, enteroids exhibit region-specific attributes of the human gastrointestinal epithelium (Middendorp et al., 2014; Saxena et al., 2016) and are non-transformed cells derived from intestinal stem cells. Enteroids have been used to examine host–microbe and host–viral interactions (Finkbeiner et al., 2012; Engevik et al., 2013; Yin et al., 2015; Saxena et al., 2016, 2017; Zou et al., 2017; Blutt et al., 2018; Rajan et al., 2018; Yin and Zhou, 2018). To the best of our knowledge, we are the first to identify folate genes in enteroids from diverse regions of the intestine. This work suggests that although the colon expresses lower levels of the major genes involved in nucleotide synthesis, methylation, one-carbon cycle, and folate transporters compared to the small intestine, it still expresses high levels of several genes including MTHFR, DHRF, MTRR, FPGS, PCFT, and RFC. Dietary FPGs are hydrolyzed to folylmonoglutamate derivatives prior to absorption by the intestinal epithelial cells. Of particular interest, the colonoids express high levels of folate hydrolase (FOLH) and γ-glutamyl hydrolase (GGH), enzymes found of the brush border and in the lysosome of the intestinal epithelial cells (Chave et al., 2003). These enzymes participate in the dietary FOLH. Additionally, only the colon expressed folate hydrolase 2 (FOLH1B), another hydrolase known to convert FPGs to folylmonoglutamates. These findings indicate that folate absorption by the human colon may significantly contribute to net folate absorption.

This study extends our knowledge of intestinal folate metabolism by using human enteroids to examine microbial folate–host interactions. Our work indicates that bacterial supernatants containing non-polyglutamylated THFs (*L. reuteri 6475:folC*-mutant strain) results in upregulation of the RFC transporter. Both PCFT and RFC are highly specific for the monoglutamate form of folate (Visentin et al., 2014). We speculate that in our enteroid model, the GCPII (folh1) cleaves the glutamate moieties to provide entrance of the *L. reuteri* folate polyglutamates into the cell. Interesting, we did not observe any changes in folh1 expression in our colonoids following bacterial supernatant treatment. Our data indicate that WT *L. reuteri*, producing a glutamylated THF and *L. reuteri 6475:folC2*, which produces no folate, had no effect on RFC levels in human colonoids. However, we do demonstrate that *L. reuteri 6475:folC*, which makes a non-glutamylated folate upregulates RFC. We reason that WT *L. reuteri’*s glutamylated folate requires extensive deconjugation by Folh1 before absorption occurs. This enzymatic deconjugation might limit the bioavailability of *L. reuteri’s* folate, thereby limiting the concentration of folate available to influence RFC levels. Since THF can be absorbed by intestinal cells (Bhandari and Gregory, 1992), we predict that the non-glutamylated folate produced by *L. reuteri 6475:folC* is taken up more rapidly and may signal to the host to upregulate the RFC receptor. Consistent with this hypothesis, the *L. reuteri 6475:folC2* mutant, which is unable to produce any folate, has no effect on RFC levels. This finding indicates that *L. reuteri* does not secrete folate-independent factors that upregulate RFC levels. Based on previously published microarray data (GSE32971), 47 of the 13,580 genes examined were downregulated in the *6475:folC* mutant compared to WT *L. reuteri* (Thomas et al., 2016). These genes included several downstream folate-related genes, including *folK* and *folB*. Additionally 64 genes were upregulated in *6475:folC*-mutant compared to WT *L. reuteri* (Thomas et al., 2016). Overall, 0.08% (111/13,580) of the *L. reuteri* genes were altered with the site-specific insertion in *folC*. We believe that this good evidence that loss of *folC* is not significantly influencing *L. reuteri*’s metabolism or metabolite profile. Additional experiments using a complemented folC in *L. reuteri* in the future would help address these questions. While we hypothesize that the glutamate status of folate influences RFC levels, it is also possible that increased RFC levels may reflect an attempt by the host to increase intracellular folate due to the lack of folate glutamylation and perhaps uptake. Currently we do not have data on microbial folate entry into the enteroid cultures. However, we believe that in the future, this enteroid culture system may serve as a valuable tool to address folate uptake questions.

Of note, cancer-derived Caco-2 cells and immortalized human colon cell line NCM 460 have demonstrated that PCFT is the primary transporter involved in intestinal folate absorption and this absorption occurs at low pH (Kumar et al., 1997; Qiu et al., 2006). While the pH of the distal colon systemically may not be favorable to folate absorption at a pH ∼7 (Evans et al., 1988; McDougall et al., 1993; Engevik et al., 2015), *L. reuteri* is known to adhere to the intestinal mucus layer directly above the epithelium and produce lactic acid which can lower the local pH (Mackenzie et al., 2010; Jensen et al., 2014). In this way, *L. reuteri* may drive additionally folate absorption in a local manner. Additionally, the cecum has a reduced pH due to the relative abundance of lactic acid bacteria and may also serve as a fertile area of folate assimilation. In our colonoid experiments, we neutralized our *L. reuteri* CM (pH = 7.0) to ensure that we did not affect our epithelial viability. In the future, varying pH parameters should be considered using the enteroid culture system.

Collectively our data indicate that the intestinal microbiome can participate in luminal folate production and subsequent modulation of folate transporter biology in the mammalian host. Selection of “smart” combinations of probiotics may provide supplemental sources of B complex vitamins such as folates, and future nutritional counseling may combine microbiome data and dietary information to guide individuals so that vitamin deficiency states are prevented. Optimizing microbial metabolism and vitamin sources in the gut microbiome may enhance human health by enhancing cellular functions and intestinal homeostasis.

## Data Availability Statement

The raw data supporting the conclusions of this manuscript will be made available by the authors upon request.

## Ethics Statement

This study (protocol H-35094) was carried out in accordance with the policies of, and approved by, the Baylor College of Medicine Institutional Review Board with written informed consent from all subjects.

## Author Contributions

MAE, CM, and JV conceived and designed the research, and drafted the manuscript. MAE, CM, DR, KE, SD, JS, and SC performed the experiments. MAE, CM, DR, KE, and JS analyzed the data. MAE, CM, and DR prepared the figures. MAE, CM, DR, KE, SD, JS, SC, MKE, MK, and JV edited and revised the manuscript. JV approved the final version of the manuscript.

## Conflict of Interest

JV serves on the scientific advisory boards of Biomica, Plexus Worldwide, and Seed Health, and he also receives unrestricted research support from BioGaia, AB.

The remaining authors declare that the research was conducted in the absence of any commercial or financial relationships that could be construed as a potential conflict of interest.

## Supplementary Material

FIGURE S1 *Lactobacillus reuteri* strains (wild-type, *6475:folC*, and *6475:folC2*) exhibit normal growth, but varying degrees of folate production. **(A)** Diagram of the genes required to convert 7,8-dihydropteroate to 5,10-methenyl-THF (tetrahydrofolate). Key genes include *folC* (purple) and *folC2* (light blue). **(B)** Growth of WT *L. reuteri* and two *L. reuteri* mutants, *6475:folC* which can produce folate, but cannot add a glutamate tail, and *6475:folC2*, which cannot produce folate, in LDM4 over 48 h. Samples were collected at 1, 3, 6, 12, 16, 24, and 48 h. **(C)** Colony forming units (CFUs) of *L. reuteri* strains at time 6, 24, and 48 h post-inoculation in LDM4. **(D)** Folate quantification in uninoculated LDM4 or WT *L. reuteri*, *6475:folC* and *6475:folC2* LDM4 conditioned media after 24 h incubation by competitive immunoassay. **(E)** Glutamylation profile of folates produced by *L. reuteri 6475:folC* determined by MALDI-TOF mass spectrometry. The diagram depicts no clear glutamate peaks for *6475:folC* folate, indicative of no polyglutamate residues. ^∗^*p* < 0.05 (*n* = 3 biological replicates, 3 technical replicates).

FIGURE S2 Expression of downstream folate targets in human colonoids treated with *L. reuteri* conditioned media. Human colon monolayers (lines Asc109 and C103) were incubated with 25% uninoculated bacterial medium (LDM4) or 25% *L. reuteri* ATCC 6475 conditioned media for 16 h. In addition to WT *L. reuteri*, the 6475:folC mutant, which can produce THF, but can’t synthesize a polyglutamate tail, and the 6475:folC2 mutant, which can’t synthesis any THF, were included in the analysis. Examination of mRNA levels **(A,E)** *folh1*, required for removing folate glutamates and **(B–H)** *methylenetetrahydrofolate dehydrogenase*, required for intracellular folate processing, by qPCR revealed no changes between groups. All qPCR data were normalized to 18S. One-way ANOVA with Bonferroni *post hoc* test (*n* = 2 biological replicates, 3 technical replicates).

TABLE S1 Bacterial strains, growth media, and exponential and stationary phase time points. ^a^LDM4 supplement: 0.1 mg/L vitamin K and 5 mg/L hemin.

TABLE S2 *Lactobacillus* defined medium 4 (LDM4) recipe.

TABLE S3 Chemically defined minimal media (CDMM) recipe.

TABLE S4 Bacterial folate synthesis qRT-PCR primers.

## Abbreviations

CHCA: α-cyano -4-hydroxycinnamic acid
CM: conditioned media
DTT: dithiothreitol
*folC2*: *folylpolyglutamate 2* gene
FPG: folylpolyglutamates
GCPII: glutamate carboxypeptidase II
GTP: guanosine triphosphate
pABA: para-aminobenzoic acid
PCFT: proton-coupled folate transporter
RFC: reduced folate carrier
SAM: *S*-adenosylmethionine
SCFAs: short chain fatty acids
THF: tetrahydrofolate.
