# Supplementary figures and images for: Microbial Metabolic Capacity for Intestinal Folate Production and Modulation of Host Folate Receptors
Source: Front Microbiol. 2019 Oct 9;10:2305. doi: 10.3389/fmicb.2019.02305 (PMC6795088; doi:10.3389/fmicb.2019.02305)

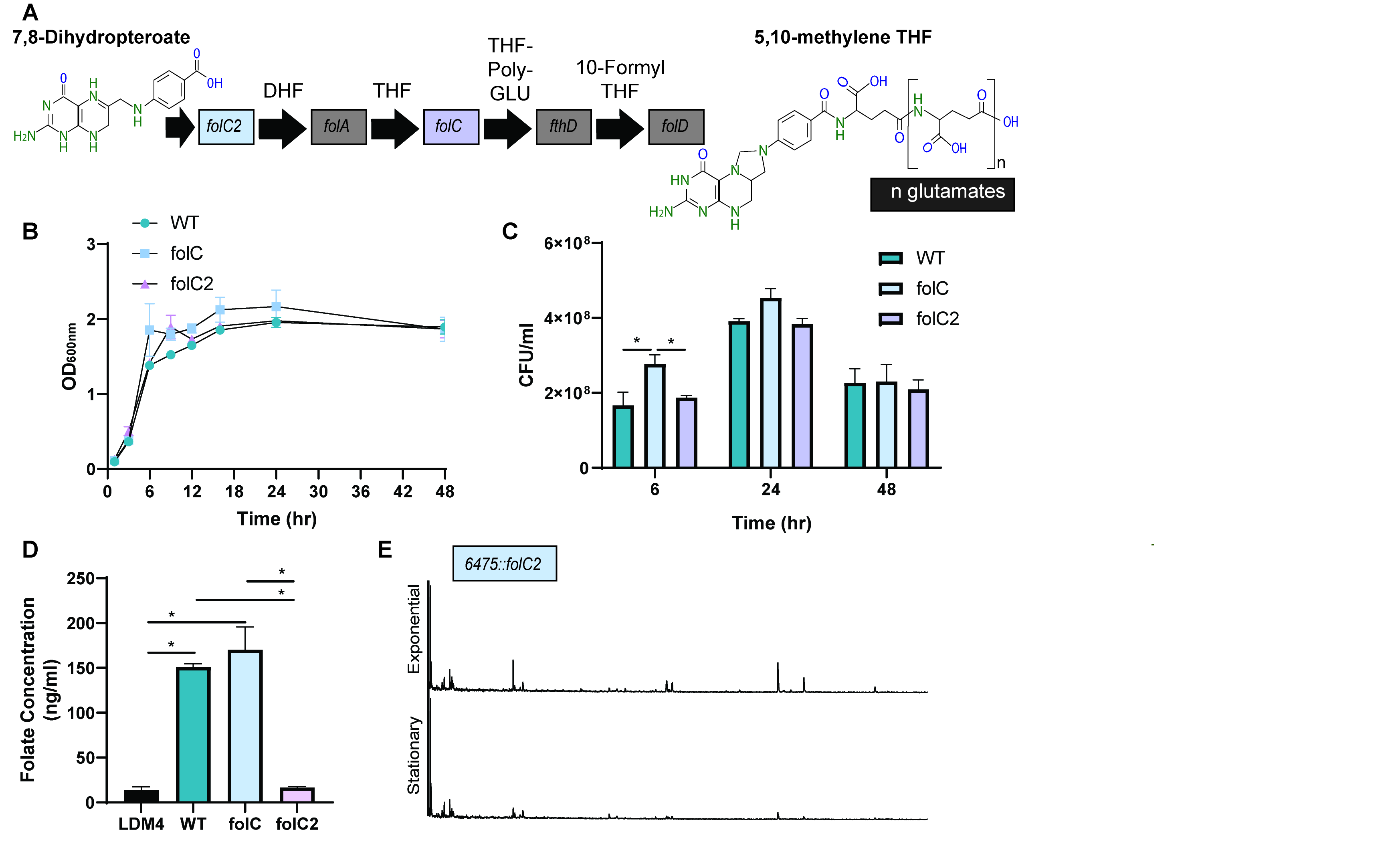

Supplement: FIGURE S1 — Lactobacillus reuteri strains (wild-type, 6475:folC, and 6475:folC2) exhibit normal growth, but varying degrees of folate production. (A) Diagram of the genes required to convert 7,8-dihydropteroate to 5,10-methenyl-THF (tetrahydrofolate). Key genes include folC (purple) and folC2 (light blue). (B) Growth of WT L. reuteri and two L. reuteri mutants, 6475:folC which can produce folate, but cannot add a glutamate tail, and 6475:folC2, which cannot produce folate, in LDM4 over 48 h. Samples were collected at 1, 3, 6, 12, 16, 24, and 48 h. (C) Colony forming units (CFUs) of L. reuteri strains at time 6, 24, and 48 h post-inoculation in LDM4. (D) Folate quantification in uninoculated LDM4 or WT L. reuteri, 6475:folC and 6475:folC2 LDM4 conditioned media after 24 h incubation by competitive immunoassay. (E) Glutamylation profile of folates produced by L. reuteri 6475:folC determined by MALDI-TOF mass spectrometry. The diagram depicts no clear glutamate peaks for 6475:folC folate, indicative of no polyglutamate residues. ∗p < 0.05 (n = 3 biological replicates, 3 technical replicates). [file Image_1.TIF]

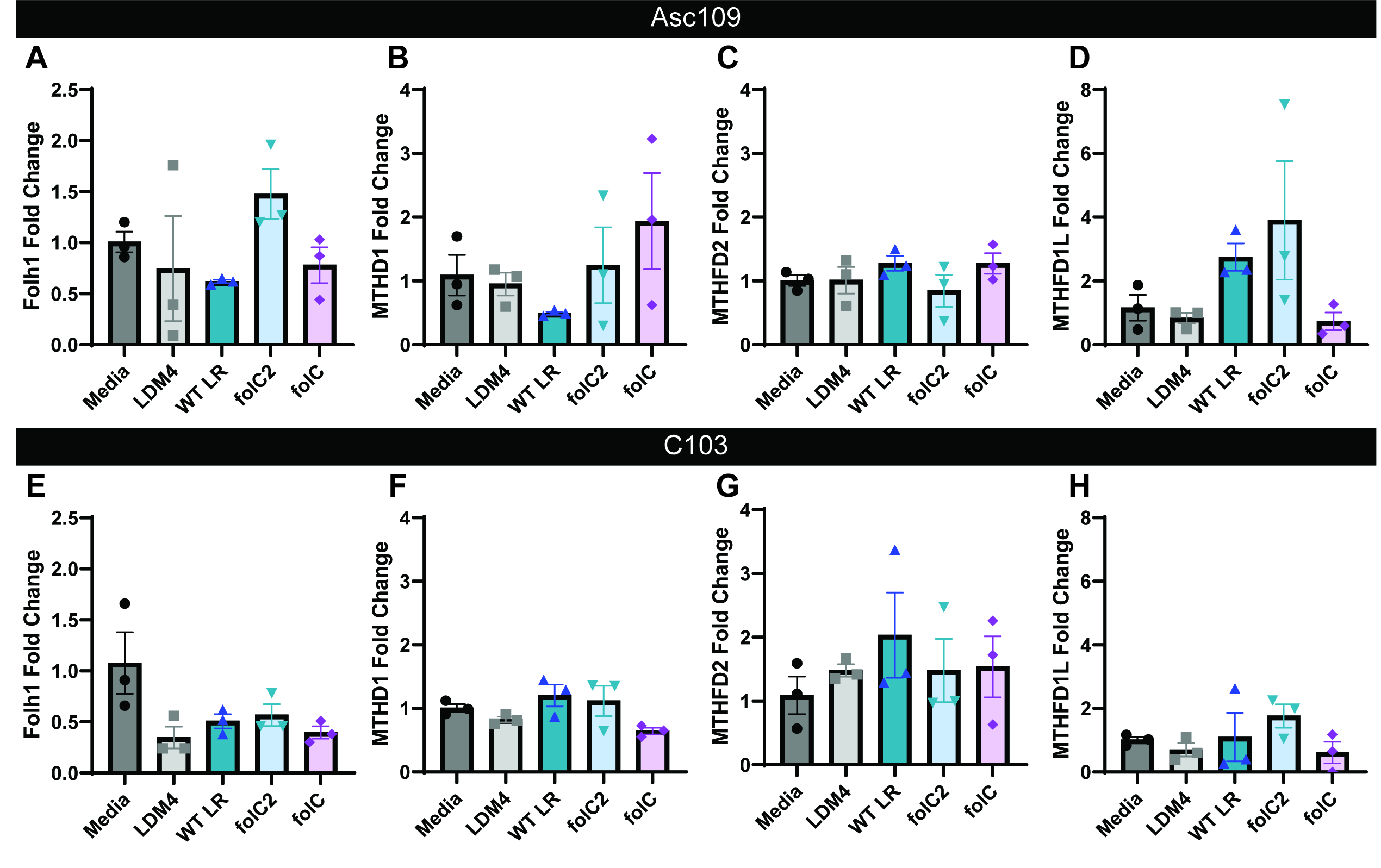

Supplement: FIGURE S2 — Expression of downstream folate targets in human colonoids treated with L. reuteri conditioned media. Human colon monolayers (lines Asc109 and C103) were incubated with 25% uninoculated bacterial medium (LDM4) or 25% L. reuteri ATCC 6475 conditioned media for 16 h. In addition to WT L. reuteri, the 6475:folC mutant, which can produce THF, but can’t synthesize a polyglutamate tail, and the 6475:folC2 mutant, which can’t synthesis any THF, were included in the analysis. Examination of mRNA levels (A,E) folh1, required for removing folate glutamates and (B–H) methylenetetrahydrofolate dehydrogenase, required for intracellular folate processing, by qPCR revealed no changes between groups. All qPCR data were normalized to 18S. One-way ANOVA with Bonferroni post hoc test (n = 2 biological replicates, 3 technical replicates). [file Image_2.TIF]
