# Supplementary material for: Microbial Metabolic Capacity for Intestinal Folate Production and Modulation of Host Folate Receptors
Source: Front Microbiol. 2019 Oct 9;10:2305. doi: 10.3389/fmicb.2019.02305 (PMC6795088; doi:10.3389/fmicb.2019.02305)
Supplement: TABLE S1 — Bacterial strains, growth media, and exponential and stationary phase time points. aLDM4 supplement: 0.1 mg/L vitamin K and 5 mg/L hemin. [file Presentation_1.pptx]

## Slide 1
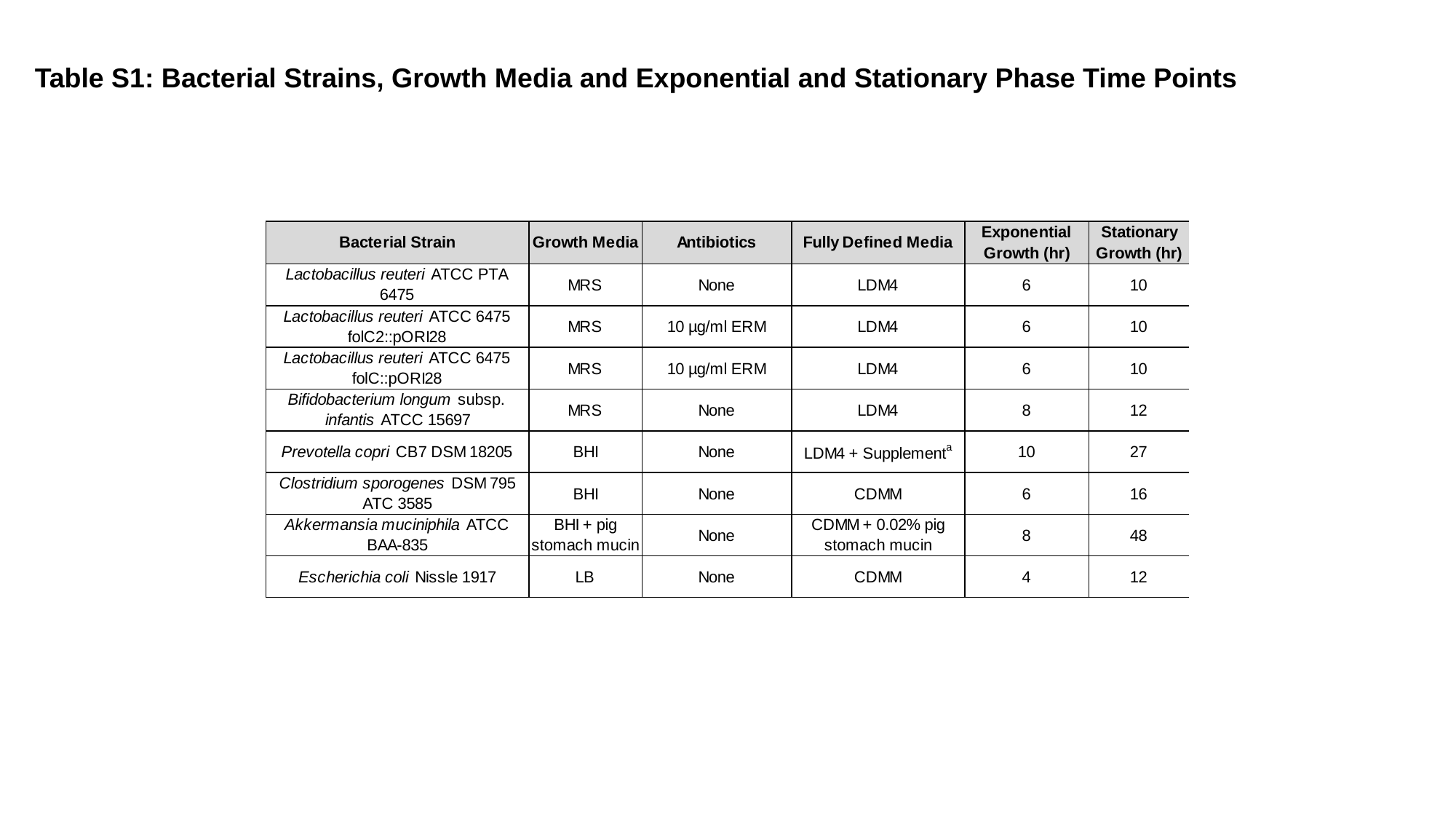

Table S1: Bacterial Strains, Growth Media and Exponential and Stationary Phase Time Points

## Slide 2
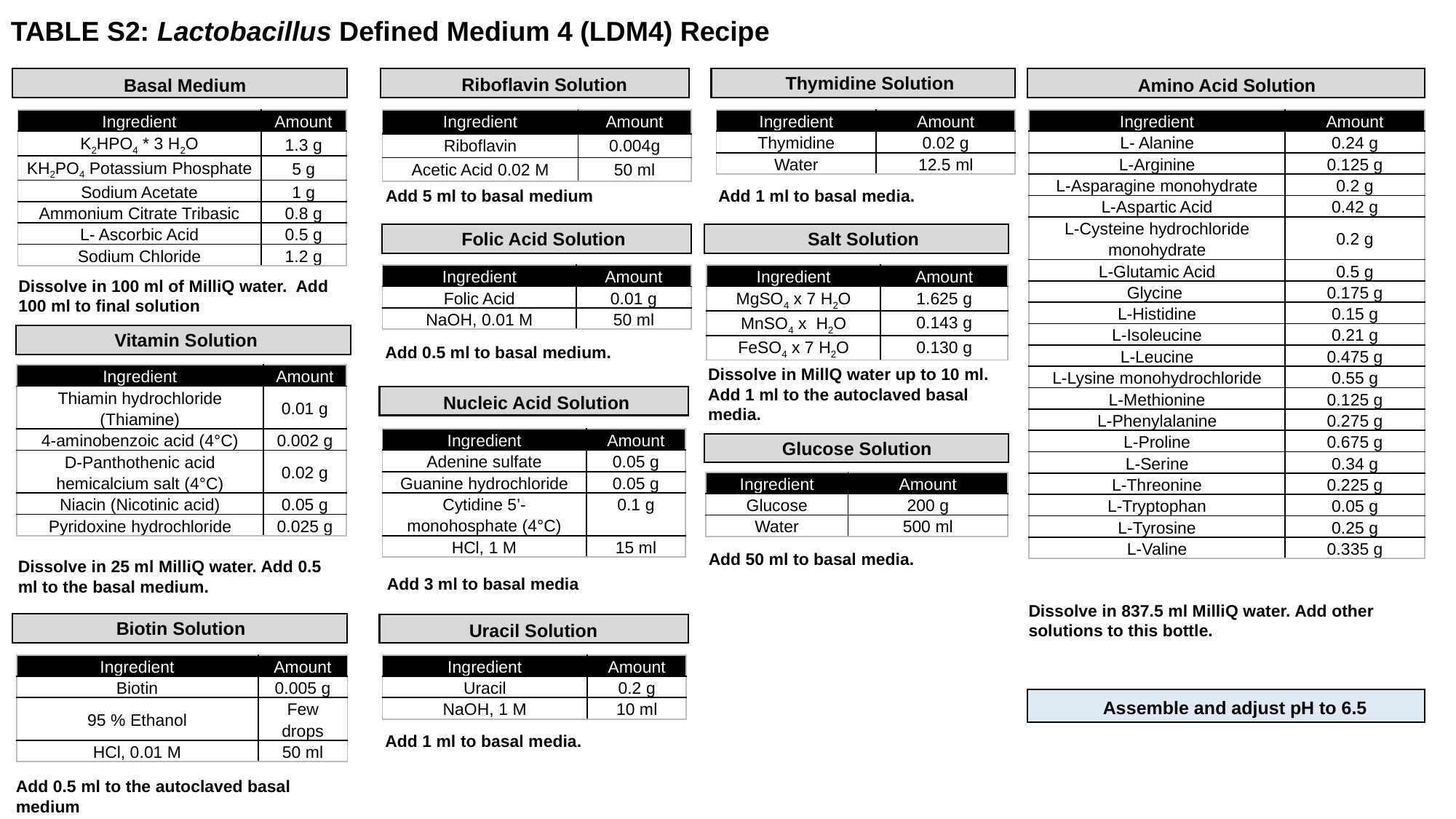

TABLE S2: Lactobacillus Defined Medium 4 (LDM4) Recipe
Thymidine Solution
Riboflavin Solution
Basal Medium
Amino Acid Solution
| Ingredient | Amount |
| --- | --- |
| Riboflavin | 0.004g |
| Acetic Acid 0.02 M | 50 ml |
| Ingredient | Amount |
| --- | --- |
| K2HPO4 \* 3 H2O | 1.3 g |
| KH2PO4 Potassium Phosphate | 5 g |
| Sodium Acetate | 1 g |
| Ammonium Citrate Tribasic | 0.8 g |
| L- Ascorbic Acid | 0.5 g |
| Sodium Chloride | 1.2 g |
| Ingredient | Amount |
| --- | --- |
| Thymidine | 0.02 g |
| Water | 12.5 ml |
| Ingredient | Amount |
| --- | --- |
| L- Alanine | 0.24 g |
| L-Arginine | 0.125 g |
| L-Asparagine monohydrate | 0.2 g |
| L-Aspartic Acid | 0.42 g |
| L-Cysteine hydrochloride monohydrate | 0.2 g |
| L-Glutamic Acid | 0.5 g |
| Glycine | 0.175 g |
| L-Histidine | 0.15 g |
| L-Isoleucine | 0.21 g |
| L-Leucine | 0.475 g |
| L-Lysine monohydrochloride | 0.55 g |
| L-Methionine | 0.125 g |
| L-Phenylalanine | 0.275 g |
| L-Proline | 0.675 g |
| L-Serine | 0.34 g |
| L-Threonine | 0.225 g |
| L-Tryptophan | 0.05 g |
| L-Tyrosine | 0.25 g |
| L-Valine | 0.335 g |
Add 5 ml to basal medium
Add 1 ml to basal media.
Folic Acid Solution
Salt Solution
| Ingredient | Amount |
| --- | --- |
| Folic Acid | 0.01 g |
| NaOH, 0.01 M | 50 ml |
| Ingredient | Amount |
| --- | --- |
| MgSO4 x 7 H2O | 1.625 g |
| MnSO4 x H2O | 0.143 g |
| FeSO4 x 7 H2O | 0.130 g |
Dissolve in 100 ml of MilliQ water. Add 100 ml to final solution
Vitamin Solution
Add 0.5 ml to basal medium.
Dissolve in MillQ water up to 10 ml. Add 1 ml to the autoclaved basal media.
| Ingredient | Amount |
| --- | --- |
| Thiamin hydrochloride (Thiamine) | 0.01 g |
| 4-aminobenzoic acid (4°C) | 0.002 g |
| D-Panthothenic acid hemicalcium salt (4°C) | 0.02 g |
| Niacin (Nicotinic acid) | 0.05 g |
| Pyridoxine hydrochloride | 0.025 g |
Nucleic Acid Solution
| Ingredient | Amount |
| --- | --- |
| Adenine sulfate | 0.05 g |
| Guanine hydrochloride | 0.05 g |
| Cytidine 5’-monohosphate (4°C) | 0.1 g |
| HCl, 1 M | 15 ml |
Glucose Solution
| Ingredient | Amount |
| --- | --- |
| Glucose | 200 g |
| Water | 500 ml |
Add 50 ml to basal media.
Dissolve in 25 ml MilliQ water. Add 0.5 ml to the basal medium.
Add 3 ml to basal media
Dissolve in 837.5 ml MilliQ water. Add other solutions to this bottle.
Biotin Solution
Uracil Solution
| Ingredient | Amount |
| --- | --- |
| Biotin | 0.005 g |
| 95 % Ethanol | Few drops |
| HCl, 0.01 M | 50 ml |
| Ingredient | Amount |
| --- | --- |
| Uracil | 0.2 g |
| NaOH, 1 M | 10 ml |
Assemble and adjust pH to 6.5
Add 1 ml to basal media.
Add 0.5 ml to the autoclaved basal medium

## Slide 3
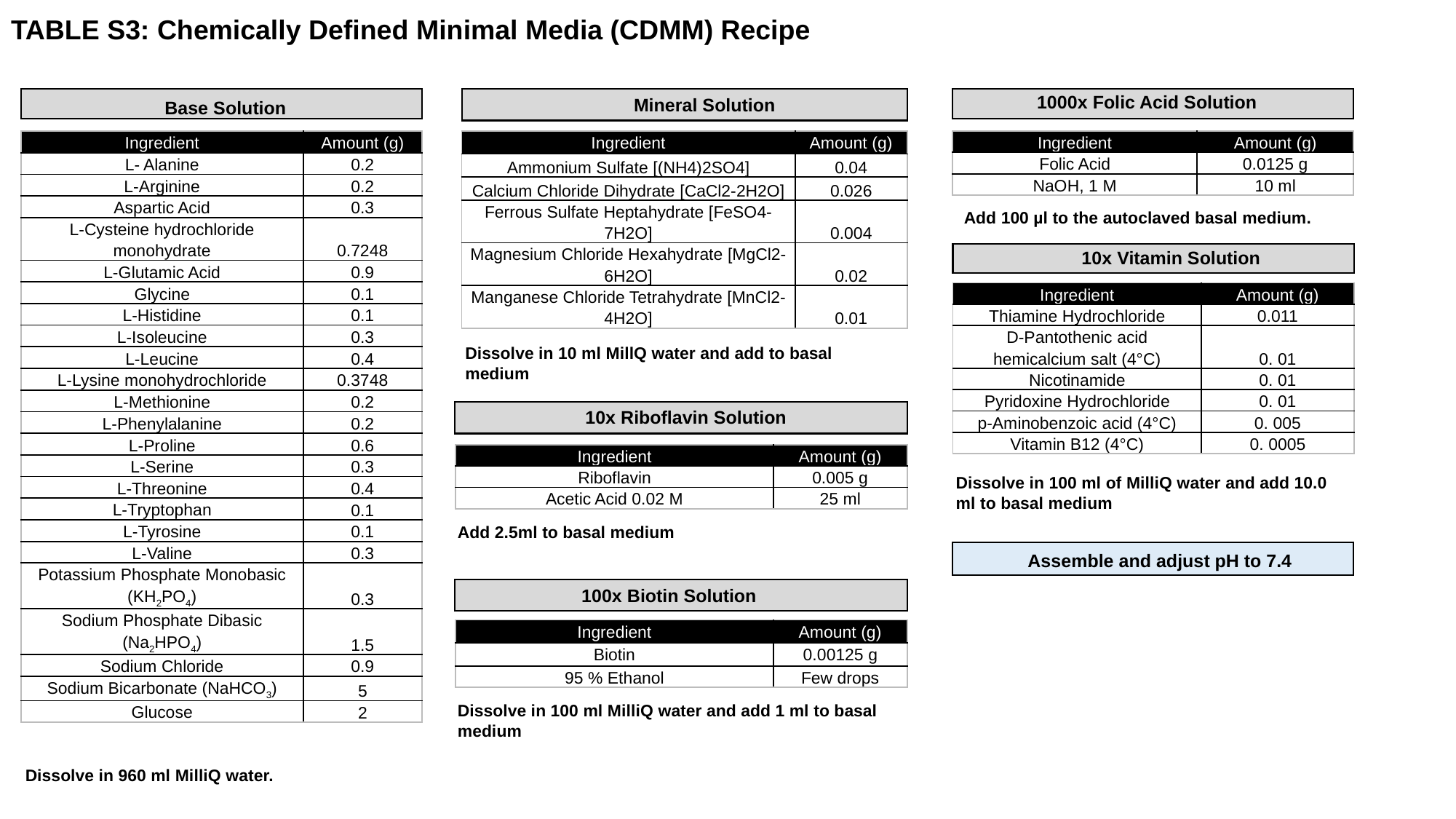

TABLE S3: Chemically Defined Minimal Media (CDMM) Recipe
1000x Folic Acid Solution
Mineral Solution
Base Solution
| Ingredient | Amount (g) |
| --- | --- |
| L- Alanine | 0.2 |
| L-Arginine | 0.2 |
| Aspartic Acid | 0.3 |
| L-Cysteine hydrochloride monohydrate | 0.7248 |
| L-Glutamic Acid | 0.9 |
| Glycine | 0.1 |
| L-Histidine | 0.1 |
| L-Isoleucine | 0.3 |
| L-Leucine | 0.4 |
| L-Lysine monohydrochloride | 0.3748 |
| L-Methionine | 0.2 |
| L-Phenylalanine | 0.2 |
| L-Proline | 0.6 |
| L-Serine | 0.3 |
| L-Threonine | 0.4 |
| L-Tryptophan | 0.1 |
| L-Tyrosine | 0.1 |
| L-Valine | 0.3 |
| Potassium Phosphate Monobasic (KH2PO4) | 0.3 |
| Sodium Phosphate Dibasic (Na2HPO4) | 1.5 |
| Sodium Chloride | 0.9 |
| Sodium Bicarbonate (NaHCO3) | 5 |
| Glucose | 2 |
| Ingredient | Amount (g) |
| --- | --- |
| Ammonium Sulfate [(NH4)2SO4] | 0.04 |
| Calcium Chloride Dihydrate [CaCl2-2H2O] | 0.026 |
| Ferrous Sulfate Heptahydrate [FeSO4-7H2O] | 0.004 |
| Magnesium Chloride Hexahydrate [MgCl2-6H2O] | 0.02 |
| Manganese Chloride Tetrahydrate [MnCl2-4H2O] | 0.01 |
| Ingredient | Amount (g) |
| --- | --- |
| Folic Acid | 0.0125 g |
| NaOH, 1 M | 10 ml |
Add 100 µl to the autoclaved basal medium.
10x Vitamin Solution
| Ingredient | Amount (g) |
| --- | --- |
| Thiamine Hydrochloride | 0.011 |
| D-Pantothenic acid hemicalcium salt (4°C) | 0. 01 |
| Nicotinamide | 0. 01 |
| Pyridoxine Hydrochloride | 0. 01 |
| p-Aminobenzoic acid (4°C) | 0. 005 |
| Vitamin B12 (4°C) | 0. 0005 |
Dissolve in 10 ml MillQ water and add to basal medium
10x Riboflavin Solution
| Ingredient | Amount (g) |
| --- | --- |
| Riboflavin | 0.005 g |
| Acetic Acid 0.02 M | 25 ml |
Dissolve in 100 ml of MilliQ water and add 10.0 ml to basal medium
Add 2.5ml to basal medium
Assemble and adjust pH to 7.4
100x Biotin Solution
| Ingredient | Amount (g) |
| --- | --- |
| Biotin | 0.00125 g |
| 95 % Ethanol | Few drops |
Dissolve in 100 ml MilliQ water and add 1 ml to basal medium
Dissolve in 960 ml MilliQ water.

## Slide 4
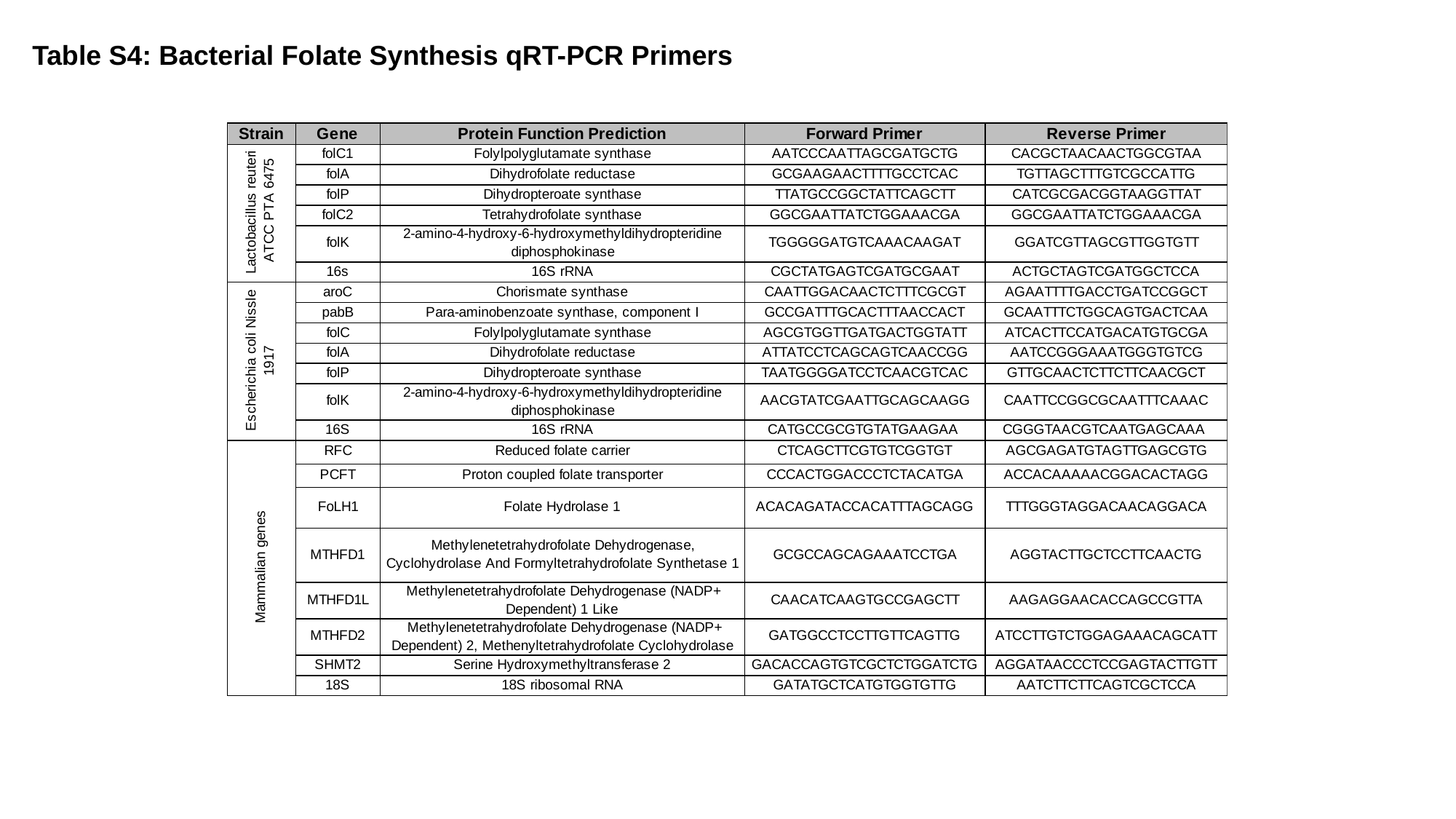

Table S4: Bacterial Folate Synthesis qRT-PCR Primers
